# Anatomy engagement and science communication

**DOI:** 10.1002/ase.70149

**Published:** 2025-11-14

**Authors:** Adam M. Taylor, Janet Philp, Kat A. Sanders

**Affiliations:** ^1^ Lancaster Medical School, Faculty of Health and Medicine Lancaster University Lancaster UK; ^2^ College of Medicine and Veterinary Medicine, University of Edinburgh Edinburgh UK; ^3^ School of Medical Sciences, Faculty of Medicine & Health University of Sydney Camperdown New South Wales Australia

Anatomy is the cornerstone of medical and allied health education, but it is also the cornerstone of life for every living person on the planet. It is the role that anatomy plays in everyone's life that underpins much of the work that has gone into this Special Issue of *Anatomical Sciences Education*. Anatomists and those who work in the Anatomical Sciences understand the intrinsic role anatomy plays in health and human life—making it a subject that inevitably captures everyone's interest at some point.

In the interconnected world we exist in today, the availability of anatomical knowledge and the precious resources that underpin it are more visible to more people than ever before. The myriad techniques that are showcased in this special edition have, in part, been developed as a result of our belief that donated human material should not be used in public engagement. Yet the work undertaken by Gomez et al.^1^ demonstrates that this is not a universally accepted concept. Examples of the challenges the anatomical community has faced continue to be drawn upon for learning and developing effective practices that meet the requirements of the present day, both inside and outside the formal learning environments. The rich history of anatomical public engagement is highlighted by Taylor and Wessel in this special issue, and we hope we have presented a balanced approach that encapsulates the many different approaches being adopted today.

Over 20 years ago Gunter Von Hagens challenged the UK anatomical community that his BODYWORLDS exhibition was a more democratic approach to anatomical education. ‘I think my exhibition, which people pay an entrance fee to see, is more democratic than anatomical education, for which the public pays through taxation but is not allowed to see’.^2^ Around the same time, Gareth Jones suggested that ‘The world of the cadaver has been deliberately shrouded in a funeral mist’ and that anatomists ‘have relied upon favorable and often vague legislation’ and this ‘lack of serious thinking about ethical issues surrounding use of the human body has left anatomists (among others) unprepared to meet the challenges of recent years’.^3^ As we hesitated, commercial companies filled the void with practices that made us shudder, linking anatomy to dubious ethical activities, reminiscent of our historical narrative of grave robbing and murder.

As anatomists we often justify our treatment of donated human remains around the concept of consent, and yet work by Chung, Zealley, and Johnson informs us that our consent process may not be as robust as we thought.^4^, ^5^, ^6^ We assume that consent is a magic concept, strengthened by the Volenti maxim—volenti non fit injuria; to one who consents, no wrong can be done^7^ and yet this has not been upheld in other, albeit extreme, cases.^8^ Wilkinson suggests that many of the restrictions that anatomists enact around access to human remains may be more a result of prudence than ethics.^9^ Disagreements over recent events such as the hotel lobby dissection^10^ and TV documentaries^11^, ^12^ are causing us to revisit our ethical stance, and this is explored further by Mussell et al.^13^ Cornwall, Jones, and Hennessey remind us that simply moving away from actual donated samples to models or images does not remove the obligation to act ethically^14^, ^15^, ^16^ particularly in the modern social media‐driven world.^17^ One only has to look at the work of the Federative International Committee for Ethics and Medical Humanities (FICEM) of the IFAA to appreciate that ethical use of our donors is still a major concern for anatomists.

Importantly, many of the projects in this issue strike a careful balance between entertainment and education, the focus of attention in Wilmshurst et al.'s paper.^18^ While engaging formats can capture attention, they must be used responsibly to ensure that learning remains central. Entertainment should be a gateway, rather than a distraction from meaningful understanding. This special issue highlights how anatomists are navigating that balance with integrity and impact.

There is also the growing role that Associations and Societies have in engagement; supporting their members, developing strategies for communication of science, combatting dis‐ and misinformation, developing guidelines and effective practice and inspiring others to learn. The role these bodies play in the strategies and leadership to support members are key to moving forward. In today's global landscape, from an engagement perspective, if we are standing still, others will already have moved on. Our Associations and Societies help build communities of practice that prioritize outreach by fostering networks, funding public engagement initiatives, and providing platforms for collaboration, as described by Dunnwald et al.^19^ They are instrumental in connecting anatomists across institutions and borders, enabling us to share resources, co‐create projects, and amplify our collective voice.

Our anatomy community may be small, but it is global, passionate, and well‐connected. Let us leverage that strength and use this issue as a springboard for action. Together, we can ensure that anatomy remains not only scientifically rigorous, but socially relevant. As time has moved forward, anatomists are once again finding themselves out of their usual educational settings and engaging with more people in more places. This special issue of *Anatomical Sciences Education* showcases a vibrant array of outreach efforts that demonstrate the creativity and commitment of our professional community. From anatomy‐focused games,^20^ to immersive virtual reality experiences,^21^ anatomists are diving beneath the skin and bringing the human body to life for learners of all ages. We hope the articles presented within give you a flavor of the vast possibilities for anatomy outreach and instill confidence to make connections. Whether you are just beginning to explore outreach or are a seasoned science communicator, we believe there is something here for everyone. Perhaps it's a method you hadn't considered, a partnership you could replicate, or a story that reminds you why you entered this field in the first place.

For those who doubt their skills in the public arena, please remember that science communication and public engagement are not separate from our traditional educational roles; they are extensions of them. The same skills we use to teach students are the same tools that, with a bit of mindful remodeling and training, make us effective communicators beyond the university. Whether through events in public spaces, school visits,^22^ podcasts,^23^ the use of engaging language,^24^ art collaborations,^25^ or museum exhibits, anatomists are uniquely positioned to make anatomy accessible and relevant to diverse audiences. These activities further enrich our teaching, deepen our understanding, and nurture much‐needed trust between science and society.

Whilst our subject may “appear” to change very little, anatomists will tell you on so many levels that no two people are the same and what inspires us is the uniqueness of anatomy. Now is the time to share that inspiration—let us be bold, be visible, and be generous with our knowledge. The public is listening, and they deserve to hear from us.

## AUTHOR CONTRIBUTIONS

**Adam M. Taylor:** Conceptualization; writing – original draft; writing – review and editing. **Janet Philp:** Conceptualization; writing – original draft; writing – review and editing. **Kat A. Sanders:** Conceptualization; writing – original draft; writing – review and editing.
